# Evaluation of photochemically cross-linked collagen/gold nanoparticle composites as potential skin tissue scaffolds

**DOI:** 10.55730/1300-0527.3521

**Published:** 2022-10-08

**Authors:** Evrim Meriç YELKUVAN, Özge ERDEMLİ, Bengi YILMAZ, Ömer AKTÜRK

**Affiliations:** 1 Department of Bioengineering, Faculty of Engineering and Architecture, Kırıkkale University, Kırıkkale, Turkey; 2 Department of Molecular Biology and Genetics, Faculty of Science and Letters, Başkent University, Ankara, Turkey; 3 Remoderm Medical Biotechnology Inc Ltd, Ankara, Turkey; 4 Department of Biomaterials, University of Health Sciences, İstanbul, Turkey

**Keywords:** Collagen type I, sericin, gold nanoparticles, green laser light, in vitro biocompatibility, skin tissue scaffolds

## Abstract

Collagen type I is the main structural unit in skin tissue and is therefore used preferentially in skin tissue scaffolds. However, collagen-based 3D scaffolds have weak aqueous stability and degradation profiles in their uncross-linked states and chemical cross-linking reagents arise toxicity concerns, which generally restrict the spectrum of their biomedical applicability. Here, the research goal is to photochemically cross-link collagen type I with rose bengal (RB) when subjected to green laser light and to investigate the effect of silk sericin-capped gold nanoparticles (S-AuNP) when incorporated into scaffolds on the cross-linking process and thus on the scaffold properties. All the collagen scaffolds, that is plain collagen (C), collagen/S-AuNP (C-Au), cross-linked collagen (C-RBL), and cross-linked collagen/S-AuNP (C-AuRBL) were characterized for their potential as skin tissue scaffolds. C-AuRBL group had the best thermal stability, resistance to enzymatic degradation, and more uniform pore size distribution. None of the groups had cytotoxicity (cell viability > 70%) regarding the microscopic observations and MTT cell viability assays for L929 fibroblasts. L929 fibroblasts and primary adult human epidermal keratinocytes (HEKa) were also separately seeded on C-AuRBL scaffolds and according to microscopy results, they could support the stimulation of adhesion, morphological changes, and spreading of both cells, thereby encouraging the usage of this fabrication strategy for prospective skin tissue scaffolds.

## 1. Introduction

Collagen type I is the underlying structural component of the extracellular matrix of the skin and it is therefore used frequently as a natural biopolymer in various skin tissue engineering applications [1–3]. Since the extracted form of collagen is lacking the required mechanical and aqueous stability, it is necessary to cross-link it to resolve these issues before exploiting it in biomedical applications [4–6]. Among the various chemical, physical, and enzymatically-induced cross-linking reactions as reviewed in the literature [7], rose bengal (RB) activated by green laser irradiation could constitute an alternative benign and efficient approach for collagen cross-linking [8–10].

Rose bengal (RB) is a photosensitizing dye molecule, which belongs to the xanthene dyes, and is attracting attention in innovative medical applications such as photochemical tissue bonding for wound closure [11], suture strengthening combinational therapy of Achilles tendon rupture [12] corneal stiffening [9], potential wound inserts for drug delivery [4] and so on due to RB-mediated protein cross-linking capability upon excitation under incident green (532 nm) laser light energy. RB has already been approved for different clinical applications [13]. The cross-linking initiation of RB on proteins was attributed to singlet oxygen (^1^O_2_) generation at higher O_2_ levels while triplet excited state RB was also claimed to have a role at lower O_2_ levels [11,14]. Different protein types were investigated for the cross-linking of RB so far such as collagen [15], human serum albumin [16], and silk fibroin [12]. Singlet oxygen formation during photochemical reactions in the presence of RB could enable the production of inter and intramolecular bonds in collagen [17]. Due to their more stable structures, increased resistance to enzymatic degradation, and enhanced biomechanical properties (i.e. increased stiffness, toughness, and tensile strength) [9, 17–20], photochemically cross-linked collagen scaffolds are expected to perform better in vitro as a skin tissue scaffold.

Gold nanoparticle-collagen nanocomposite structures were fabricated in forms like sponges [1], nanofibrous mats [21], gels [22, 23] so on, and have already been reported in the review articles [24] and book chapters [25] to have beneficial features as skin tissue scaffolds, which could be briefly listed as enhanced mechanical, structural, aqueous stability, and wound healing ability.

Sericin, a degumming protein isolated from the cocoons of *Bombyx mori* (*B. mori*) silk, was exploited in skin tissue scaffolds for their inherent cytoprotective and mitogenic effects [26], L929 cell proliferative, antiinflammatory, antioxidant, and wound healing promoting effects [27]. Sericin could be complexed with AuNPs to enhance the aqueous stability [28] and improve in vitro biocompatibility in L929 cell lines, and antibacterial activity for streptomycin [29], and also have catalytic action on the reduction of methylene blue [30].

**Figure 1:**
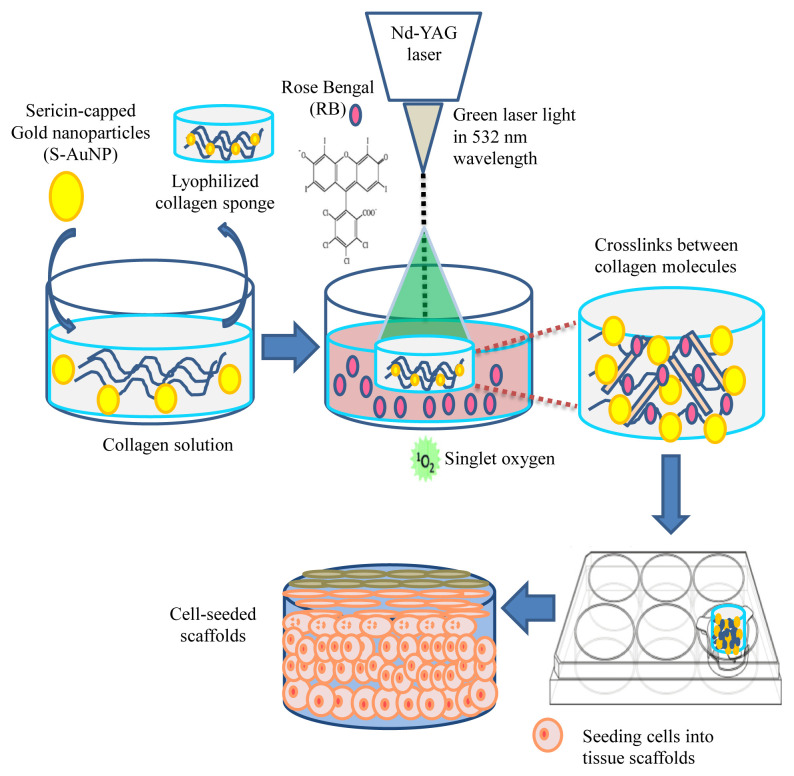
A schematic showing the fabrication steps of skin tissue scaffolds.

The hypothesis of this study is to explore the potential of collagen/sericin-capped gold nanoparticle scaffolds for photochemical cross-linking initiated by RB and laser irradiation (RBL). Hence, the research goal here is to achieve the collagen cross-linking by this RBL process first and then to test and confirm their enhanced stability and biological activity as potential skin tissue scaffolds. After the scaffold fabrication and testing the structure’s robustness against aqueous, thermal degradation and the suitability of the 3D porous architecture of the scaffold as a skin tissue scaffold, cell-scaffold interaction was investigated by seeding L929 fibroblasts or normal adult human epidermal keratinocytes (HEKa) into the scaffolds. The cytotoxicity of scaffolds in L929 fibroblasts was initially evaluated with MTT cell viability tests and light microscopy observations, then the morphology and spreading of cells were observed with scanning electron microscopy (SEM). The fabrication methodology was summarized with a schematic (Figure 1).

## 2. Materials and methods

### 2.1. Materials

Collagen type I (from bovine Achilles tendon), rose bengal, absolute ethanol, collagenase type 1 enzyme, glutaraldehyde, phosphate-buffered saline tablets, dimethyl sulfoxide (DMSO, for molecular biology), and MTT [3-(4,5-Dimethyl-2-thiazolyl)-2,5-diphenyl-2H-tetrazolium bromide] were purchased from Sigma-Aldrich (Germany). ThinCert™ cell culture insert was bought from Merck (Germany). Epilife^®^ medium with 60 μM calcium chloride and the human keratinocyte growth supplement (HKGS) was purchased from ThermoFisher Scientific (USA). Dulbecco’s Modified Eagle Medium (DMEM with 4.5 g/L D-glucose), fetal bovine serum (FBS), penicillin-streptomycin-Amphotericin B solution, and trypsin/EDTA solution A with phenol red were bought from Biological Industries (USA). Other cell culture reagents and disposables were obtained from Biological Industries (USA), or else the required specifications were given in the text.

### 2.2. Synthesis of sericin-capped gold nanoparticles by laser ablation

Silk sericin of *B. mori* origin, isolated and chemically specified in our previous publication [29], was dissolved in 5 mL of deionized water (at pH 4, 7, 10, and 14 conditions) to get a solution at 0.25% (w/v) concentration. A pure gold plate (24-carat purity, Kırıkkale, Turkey) was put inside a beaker containing the above sericin solution (5 mL) and ablated with Nd-YAG laser apparatus (Model: AML-1201, China) as mentioned in detail in a recent study [31]. Briefly, sericin-capped gold nanoparticles (S-AuNP) were synthesized with the optimized laser parameters (frequency = 10 Hz, pulse time = 10 ns, pulse energy = 1000 mJ, wavelength = 532 nm, the laser count number = 1000 times) and S-AuNP amount released into sericin solution was determined gravimetrically as 0.6 mg. The control AuNP group (with no sericin) was synthesized similarly for stability studies.

For the characterization of S-AuNP, initially, the surface plasmon resonance (SPR) peaks were identified spectrophotometrically (Biochrom Libra S70 Spectrophotometer, Biochrom Ltd., United Kingdom) by scanning the spectra of AuNP suspensions in the 280–900 nm wavelength range. The zeta potential and size distribution measurements were done with a Zetasizer (Nano ZS, Malvern Instruments, Malvern, England). The size, shape, and size distribution of S-AuNP sample suspensions were viewed with a High Contrast Transmission Electron Microscope (CTEM, 120 kV, Tecnai^™^ G^2^ Spirit Biotwin, FEI). The size of S-AuNP in grid regions (n ≥ 300) was evaluated through an image analysis program (Image J, NIH, USA), thus the mean nanoparticle size was calculated and size distribution analysis was carried out by plotting a histogram in equally-sized span values. Both a % size-frequency bar graph and a % cumulative curve were given in this histogram and span values were also calculated from the % cumulative curve to show the size dispersion as follows:


(1) $$Span=\frac{d[0.9]-d[0.1]}{d[0.5]}$$

where d[0.9], d[0.5], and d[0.1] are the particle diameters determined at the 90th, 50th, and 10th percentile of undersized particles, respectively. Regarding the stability, sericin-capped AuNP obtained in different pH conditions were incubated in buffers containing different salt concentrations, and the SPR peak changes were observed with a spectrophotometer (PowerWave XS2 Microplate Spectrophotometer, BioTek, USA) to evaluate the stability as similar to our previous studies [29, 31].

### 2.3. Fabrication of skin tissue scaffolds

The dissolution protocol of the main scaffolding material, collagen type I, was executed similarly to our previous study [1]. In brief, 1% concentration was obtained by dissolving the lyophilized collagen in 3% acetic acid. While being held in an ice-cold water bath the collagen solution was stirred and then homogenized to get a fibrous suspension. This procedure was repeated until obtaining a homogenous suspension. The 1% collagen solution was mixed with S-AuNP solution at a 4:1 volumetric ratio and the mixture (10 mL) was poured into a glass Petri mold (Group C-Au). The control group without S-AuNP (Group C) was prepared by considering this volumetric ratio with the addition of deionized water in place of S-AuNP. Finally, the solutions in molds were frozen at −80 °C and lyophilized in a freeze-dryer (approximately at −40 °C chamber temperature and 0.07 mbar vacuum pressure conditions, Christ Freeze Dryer, Alpha 1–2 LD Plus, Germany) to get spongy scaffolds after 1 day. For the photochemical cross-linking process, the obtained scaffolds, C and C-Au were soaked in rose bengal (RB) solution (0.1% w/v in 100% ethanol), and exposed to laser irradiation in a dark environment from both bottom and top surfaces (500 counts, 532 nm wavelength, 1000 mJ pulse energy, 10 Hz pulse frequency). The codification of cross-linked groups was: laser-treated collagen sponge soaked in RB (Group C-RBL) and laser-treated collagen/S-AuNP blend soaked in RB (Group C-AuRBL). After this cross-linking procedure, C-RBL and C-AuRBL were also subjected to UVC light irradiation of 2 parallelly positioned UVC lamps 30 cm apart from the scaffold surfaces (254 nm, 15 W, OSRAM PURITEC HNS Germicidal Lamp, Russia) from both sides, each for 30 min. The unreacted RB residuals in C-RBL and C-AuRBL were rinsed thoroughly with graded levels of ethanol series (20% decrements) so as not to disturb irreversibly the delicate structure of scaffolds and eventually rehydrated in 100% water. Finally, the scaffolds were frozen at −80 °C and freeze-dried again (the same process mentioned above). All the dried-out scaffold groups were stored in a desiccator placed in a +4 °C freezer until the experiments.

### 2.4. Morphology analysis by scanning electron microscopy

The morphology of the scaffolds was examined by Field Emission Scanning Electron Microscopy (FE-SEM, QUANTA 400F, FEI, USA) with an acceleration voltage of 20 kV. Before the analysis, the samples were coated with a thin layer of gold-palladium (Au-Pd) and micrographic images of the scaffolds from the top and cross-sections were taken to observe the morphologies.

### 2.5. Pore size distribution

The porous structure of skin tissue scaffolds in terms of their pore size distribution was analyzed with a Mercury Porosimeter (Poremaster 60, Quantachrome Corporation, USA) in low-pressure mode (0–50 psi). The results were plotted as bar graph histograms as pore volume (normalized by scaffold mass) vs. pore diameter (μm).

### 2.6. Attenuated total reflectance-Fourier transformed infrared spectroscopy

The chemical functional groups of C, C-Au, C-RBL, and C-AuRBL scaffold groups were analyzed with attenuated total reflectance-Fourier transformed infrared spectroscopy (ATR-FTIR) (IFS/66S, Hyperion 1000). Each spectrum was obtained with a ZnSe ATR crystal cell with a total of 256 scans in transmittance mode of 4 cm^−1^ resolution and a spectral range of 4000–400 cm^−1^

### 2.7. In vitro degradation

The aqueous stability of scaffolds was evaluated with PBS-buffered media alone (hydrolytic degradation) or PBS-buffered solution supplemented with a collagenase type I enzyme (enzymatic degradation) as shown previously [1]. Briefly, for hydrolytic degradation (HD), the samples cut out at 1 cm × 1 cm were placed in PBS (pH: 7.2, 0.01M, 5 mL) and incubated at 37 °C as long as 10 days, while for the enzymatic degradation (ED) the same-sized samples in the PBS were added 60 μL of collagenase type I solution from stock (1 mg/mL, in d-H_2_O) and incubated similarly until the sampling times. The sampling times of groups were selected as 5 and 10 days for HD whereas they were 1, 4, and 8 h for ED until the complete disruption of scaffold integrity. For the calculations, scaffolds were removed from the incubation media and dried out to measure the dry masses gravimetrically by digital balances. The degradation results were calculated by using the following equation:


(2) $$Degradation\;(\%)=\frac{W_{i}-W_{t}}{W_{t}}\times 100$$

Where W_i_ and W_t_ indicated dry masses of test samples before (initial) and after an incubation time t, respectively.

### 2.8. Thermogravimetric analysis

Thermogravimetric analysis was conducted using Thermogravimetric Analyzer (TGA) (Perkin Elmer Pyris 1, USA) to determine the % weight loss of tissue scaffolds as a function of temperature. The scaffolds were weighed (20 mg) before transferring them into platinum pans. The weight changes of scaffolds were monitored while heated from the temperature of 20 °C to 950 °C under a nitrogen atmosphere at a heating rate of 30 °C/min to characterize their thermal decomposition responses.

### 2.9. Differential scanning calorimetry

The thermal stability of the skin tissue scaffolds was analyzed by differential scanning calorimetry (PerkinElmer Diamond, USA). Five mg test samples were weighed with digital balance and sealed in standard aluminum pans. An empty pan was used as a reference. The thermograms were obtained in an N_2_ atmosphere with a flow rate of 20 mL/min through sequential heating and cooling cycles from 20 °C to 300 °C with 10 °C/min heating and cooling rates.

### 2.10. Dynamic mechanical analysis

The viscoelastic behavior of tissue scaffolds was evaluated by using a Dynamic Mechanical Analyzer (DMA, Perkin Elmer Pyris Diamond, USA) at a heating rate of 2 °C/min sweeping from 25 °C to 300 °C with a constant frequency of 1 Hz and 0.05% strain. The thermomechanical properties such as storage modulus (E′), loss modulus (E′), and damping factor (tanδ = E′/E″) were measured with triplicate sampling.

### 2.11. Cell culture, cytotoxicity, and cell seeding studies

L929 mouse fibroblast cell lines (ATCC^®^ CCL-1™, USA) routinely utilized in cell culture studies were selected for the cytotoxicity evaluation of all test groups before keratinocyte cell seeding experiments. Cryopreserved Primary Human Epidermal Keratinocytes, adult (HEKa, Cat No: C-005-5C) were purchased from ThermoFisher Scientific (USA). The frozen vial containing HEKa was stored in the vapor phase of a liquid nitrogen freezer upon receipt, and then the thawing and culturing protocol was applied concerning the related manual document of GIBCO Invitrogen cell culture. Briefly, the whole content of the 500 mL Undefined Epilife^®^ medium was mixed with 5 mL of HKGS. Hence, the final concentrations of the components in the supplemented Epilife^®^ medium became: 0.2% v/v bovine pituitary extract (BPE), 0.01 μg/mL recombinant human insulin-like growth factor-I, 0.18 μg/mL hydrocortisone, 5 mg/mL bovine transferrin, and 0.2 ng/mL human epidermal growth factor. L929 or HEKa cells were subcultured until reaching 80% confluency and then were detached for passaging or cell seeding onto scaffolds with trypsin/EDTA (0.025%: 0.01%). The detached floating cells were counted with an automatic hemocytometer (Countess™ II FL Automated Cell Counter, ThermoFisher Scientific, USA) for determining the cell density.

Cytotoxicity tests were designed by complying with ISO 10993-5 standards [32]. Before the tests, the scaffolds (1 cm × 1 cm) were first sterilized in 70% ethanol supplemented with 1% penicillin-streptomycin overnight and afterward exposed to UVC light in a safety cabinet for 30 min. Finally, the sterilized samples were rinsed with PBS thoroughly to get rid of any ethanol residuals. L929 cell seeding density was adjusted as 3 × 10^4^ cells/well inside each well of a 12-well plate. After a 1-day incubation period in a humidified 5% CO_2_ incubator at 37 °C, the media was refreshed and the test specimens (that is the C, C-Au, C-RBL, and C-AuRBL) were directly placed onto the cell monolayer floating in the cell culture media and incubated again for 1 and 3 days. Initially, a qualitative assessment of cytotoxicity was made by microscopic observations of cell morphology as compared with negative control (untreated one). For a quantitative assessment, after the incubation, the media inside each well was removed and replaced with methyl thiazolyl diphenyl tetrazolium bromide (MTT) working solution (0.5 mg/mL, dissolved in phenol-free DMEM). After an incubation of 3 h in a dark environment, the forming formazan crystals were solubilized with 0.1 mL DMSO and the plate was subjected to 200 rpm agitation in an orbital shaker for 5 min. Then, spectrophotometric measurements were made with an ELISA microplate reader (PowerWave XS2 Microplate Spectrophotometer, BioTek, USA) at 570 nm wavelength. The sample OD readings were normalized by the OD of negative control and hence % cell viability values were calculated (n = 3).

L929 and HEKa cells were seeded on the C-AuRBL scaffolds and incubated for 1 day, and then fixed with 3% glutaraldehyde. Finally, they were kept at −80 °C until the freeze-drying process. The freeze-dried cell-seeded scaffolds were examined by SEM (FE-SEM, QUANTA 400F, FEI, USA). For the preparation of SEM samples, cell-seeded scaffolds were first mounted on stubs, then they were exposed to a sputter-coating period finalizing with a gold/palladium (Au/Pd) thin-film surface coverage.

### 2.12. Statistical analysis

One-way Analysis of Variance (ANOVA) tests were applied first to all test groups to make a statistical comparison and then t-tests (assuming equal variances) were utilized to determine whether there exists a significant difference between dual groups (MS Excel package program). A p-value less than 5% was accepted as statistically significant (p < 0.05). The data were expressed as mean ± standard deviation (SD) on the plots.

## 3. Results and discussion

### 3.1. Characterization of sericin-capped gold nanoparticles

**Figure 2:**
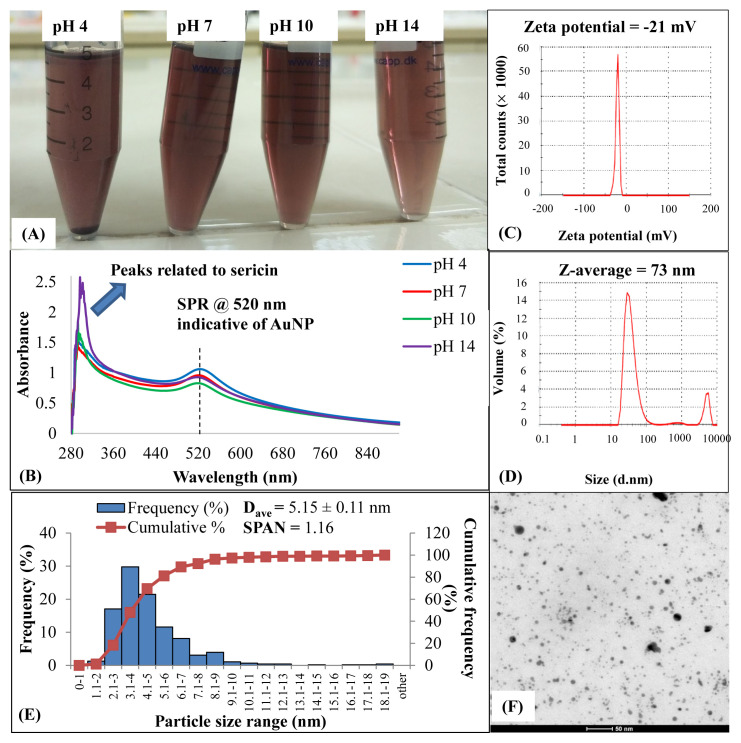
Aqueous stability response against pH changes, Zetasizer results, and size analysis by TEM of laser-ablated gold nanoparticles synthesized in a medium containing 0.25% silk sericin: (A) the photos demonstrating the color change, (B) OD spectra scans, (C) zeta potential measurements (pH 7), (D) size distribution by volume analysis (pH 7), (E) particle size distribution histogram, and (F) representative TEM image (pH 7).

**Figure 3:**
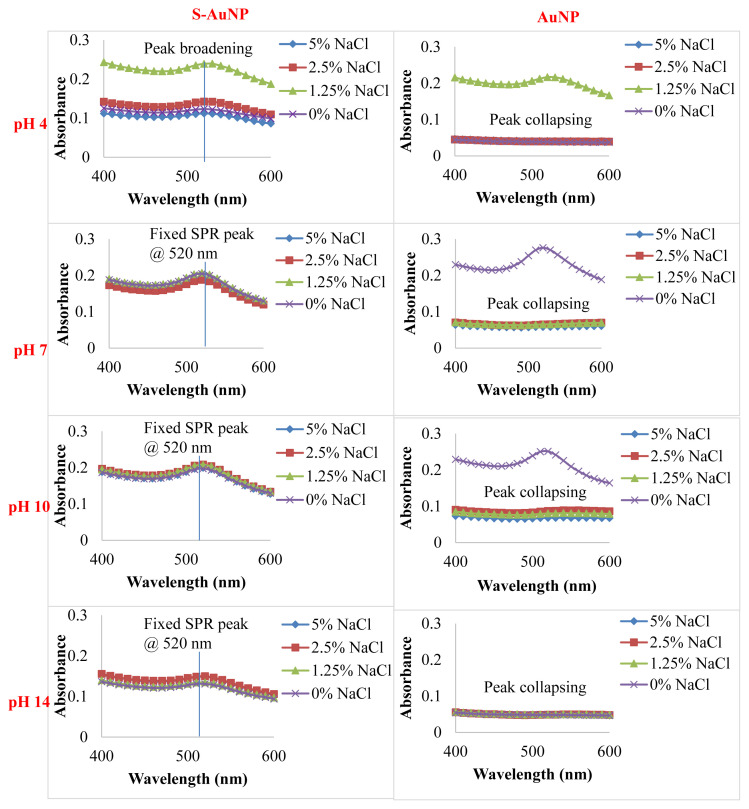
Aqueous stability results of sericin-capped AuNP (S-AuNP) and only AuNP (as a control group) tested in buffers containing different NaCl concentrations.

The formation of AuNP in experimental groups containing silk sericin (0.25% w/v) at different pH values was confirmed both by ruby red color changes and SPR peak observations in UV-Vis spectra (Figure 2A and 2B). Redshifts in the spectra (SPR shifts towards the larger wavelengths) were an indication of bigger AuNP in suspensions of pH 4 while on the contrary, the blue shifts in other groups signified the presence of smaller AuNP in comparison (Figure 2B). Here the SPR peaks were detected at around 520 nm, which is characteristic of AuNP in the literature [31], and this initial observation confirmed the success of the AuNP synthesis reaction. Regarding the intensity of the SPR bands for AuNPs, they tended to diminish as the pH values increased; however, these decrements were not significant and their spectra shapes were nearly the same. Also, no additional SPR peaks emerged when the pH changed from the neutral condition as can be verified in the full UV-Vis spectral scans. The SPR peaks, especially at pH 7 and 10, were more distinct, which also pointed out a more concentrated nanoparticle formation. In contrast, nanoparticle formation was suppressed in other pH groups. The solubility of silk sericin in an acidic pH 4 was confirmed to be poor by the sediments at the bottom of tubes during the dissolving process whereas sericin dissolves completely in basic solvents (Figure 2A). The presence of excess sericin in the synthesis medium (the pH 14 group) was thought to avoid nanoparticle formation by a mechanism of steric hindrance. The signature of sericin in AuNPs was encountered in the 280–300 nm wavelength region in UV-Vis spectral analysis (Figure 2B). As the pH increased to the alkaline region, the peaks intensified directly proportional to the increased solubility of sericin. According to ZetaSizer results, the S-AuNP group had negative zeta potential (Figure 2C) and 73 nm hydrodynamic diameter in the analysis of size distribution by volume (Figure 2D). In Figures 2E and 2F, the histograms of particle size distributions indicated a normal distribution with a mean particle size of 5 nm and a span value of 1.16. This span value greater than 1 was due to the presence of bigger AuNPs (until 19 nm). TEM figures demonstrated that there were dominantly spherical AuNPs in segregated regions of TEM grids, which could also provide proof of the stabilizing effect of sericin by preventing nanoparticle aggregation. To confirm further the stabilizing effect of sericin for AuNPs, the S-AuNP and AuNP groups were challenged against different pH and NaCl conditions (Figure 3) and the results suggested that sericin indeed helped to maintain the stability of AuNPs in the aqueous environment even in the worst scenario tested (pH 4 and 5% NaCl conditions) to an extent with some peak broadening indicative of AuNP aggregation [29, 31]. On the contrary, for the control AuNP group with no sericin capping, SPR peaks tended to collapse completely for all pH conditions while NaCl existed. The peak broadening and the loss of SPR peaks are clear signs of disruption for AuNP stability as reported previously [29,31]. The best pH conditions for S-AuNP stability were also determined to be at 7 and 10 values from the analysis, in contrast, the worst one was pH 4.

### 3.2. Characterization of skin tissue scaffolds

**Figure 4:**
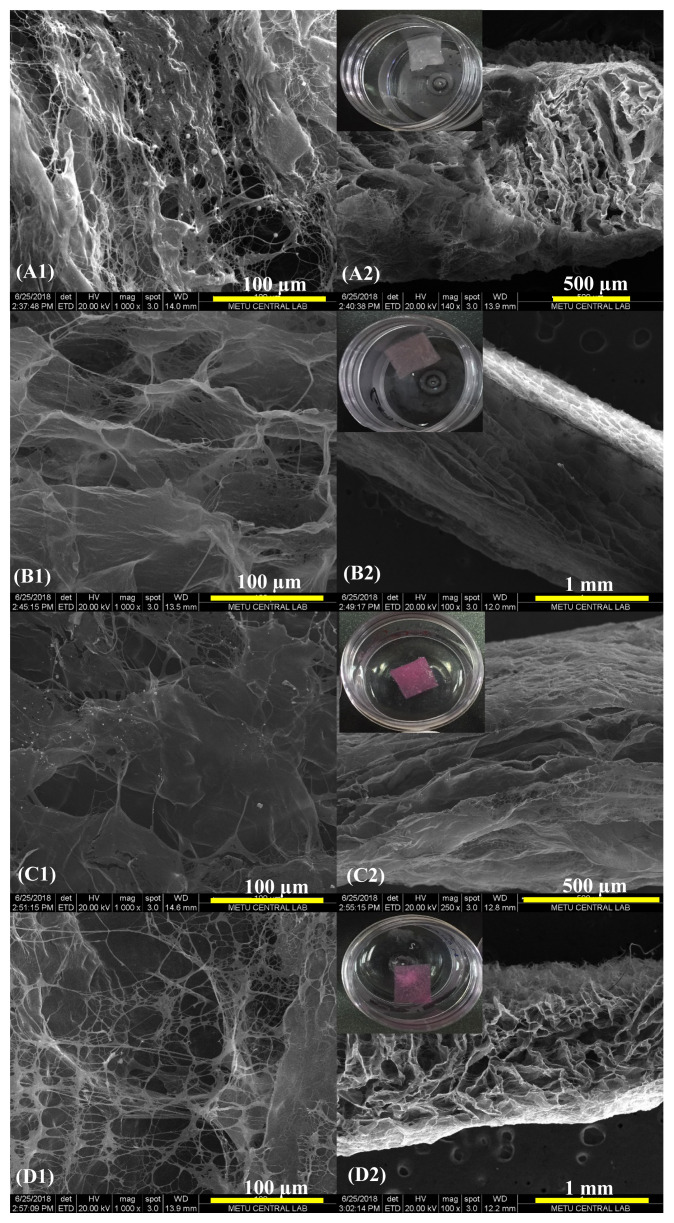
SEM micrographs of different collagen scaffold groups from the top and side views (inset figures are the corresponding photos of scaffolds): (A1 and A2) C, (B1 and B2) C-Au, (C1 and C2) C-RBL, and (D1 and D2) C-AuRBL.

**Figure 5:**
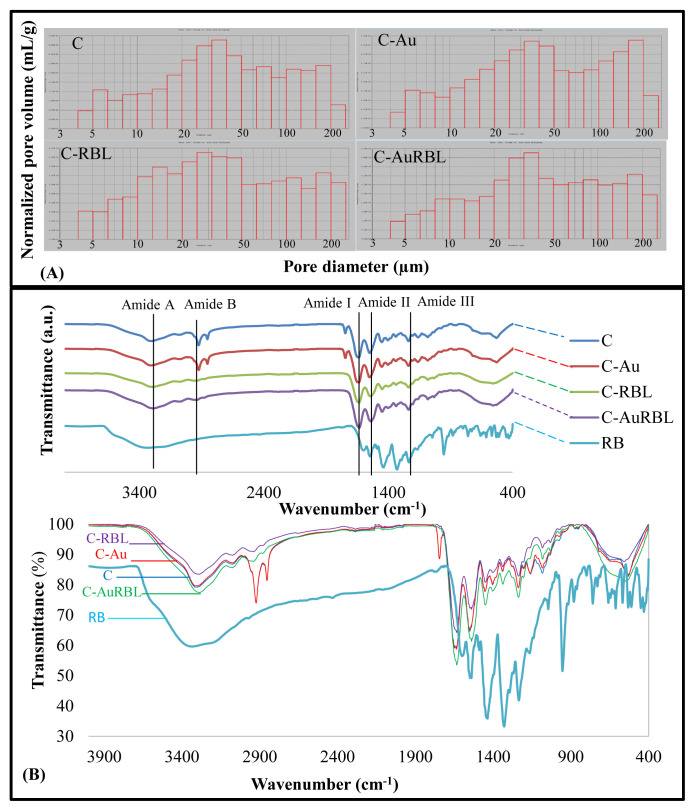
(A) Pore size distribution and (B) FTIR spectra analysis of different scaffold groups (the bottom is superimposed spectra).

The morphology of scaffolds was examined from top and cross-section views by Scanning Electron Microscopy (Figure 4). Untreated scaffold groups (C and C-Au) had spongy forms with abundant pores that had large sizes (Figures 4A and 4B). This porous structure was preserved to a great extent after the photochemical cross-linking treatment (Figures 4C and 4D). Especially in the groups containing AuNP, a more porous structure with a homogenous pore size distribution was observed (Figures 4B and 4D). The changes in the 3-dimensional structure of samples after the treatments and the interconnectivity of pores were examined through cross-sectional views (Figures 4A2–4D2). There were no considerable changes in the 3D structures after the treatments. The thicknesses of scaffolds varied between 0.5–1 mm. The thicknesses (and thus the 3D morphology) of C-RBL and C-AuRBL changed due to the vigorous rinsing and lyophilization procedures applied after the photochemical cross-linking treatment. Although these treatment groups preserved their shapes and thicknesses in the photochemical cross-linking process, they tended to collapse to an extent during the rinsing. The red color of scaffolds after the treatment is the characteristic color of RBL. The pore size distribution of all spongiform scaffolds was examined by Mercury Porosimeter at low pressure (Figure 5A). All the collagen-based scaffolds had a wide pore size distribution in between 5–200 μm sizes and the groups had resemblance in this perspective. However, in the C-Au group, the proportion of larger-sized pores was slightly greater than the others. As regards the C-AuRBL group, the pores concentrated especially in the size range of 30–40 μm.

FTIR spectra of different test groups are given in Figure 5B. All the characteristic peaks which are peculiar to collagen type I were observed clearly. The positions of peaks and bands specific to collagen were listed as 1634–1635 cm^−1^ (N-H bending vibrations coupled to C = O stretching vibrations stemming from amide I band), 1539–1549 cm^−1^ (C-N stretching vibrations coupled to N-H bending vibrations stemming from amide II band), 1236–1237 cm^−1^ (N-H deformation and C-N stretching vibrations combinational peaks stemming from amide III band) [33]. Amide A and Amide B peaks were identified at 3301–3287 cm^−1^ (due to O-H and N-H stretching vibrations) and 3000–2800 cm^−1^ (C-H stretching vibration of CH_2_ group), respectively [34]. The peaks emerging at 1453–1449 cm^−1^ were related to the stereochemistry of the pyrrolidine rings [34]. The collagen triple helix structure was confirmed to be intact by calculating the peak intensity ratios of amide III and 1453–1449 cm^−1^ bands as close to 1 [34]. By using this logic, the triple helix nature of the collagen after crosslinking was determined to preserve its integrity, because the peak absorbance ratios (1236 cm^−1^/1449 cm^−1^) were calculated to be 0.9 for both C-RBL and C-AuRBL groups. No significant differences in the FTIR spectra were observed for C and C-Au groups since the AuNPs were seemingly not involved in any chemical reactions before RBL treatment. On the other hand, the locations (shift to the lower wavenumbers) and intensity (decrease for C-RBL but increase for C-AuRBL) of the amide A and amide II bands in both C-RBL (3288 cm^−1^ and 1539 cm^−1^) and C-AuRBL (3287 cm^−1^ and 1539 cm^−1^) groups changed explicitly. Likewise, for these groups, the peak pairs at 3000–2800 cm^−1^ that belong to C-H stretching vibrations of amide B changed drastically in intensity (almost completely lost) and increased in wavenumber. The intensity of the amide I band also changed for C-RBL (decrease) and C-AuRBL (increase), but the positions were fixed. These changes indicated the cross-linking of collagen structures in the presence of rose bengal via these chemical functional groups. By taking a glance at the literature data [11,35], it was stated that the carboxyl group of rose bengal and the amino group of the collagen chemically interacted to trigger a cross-linking event in the protein structure.

**Figure 6:**
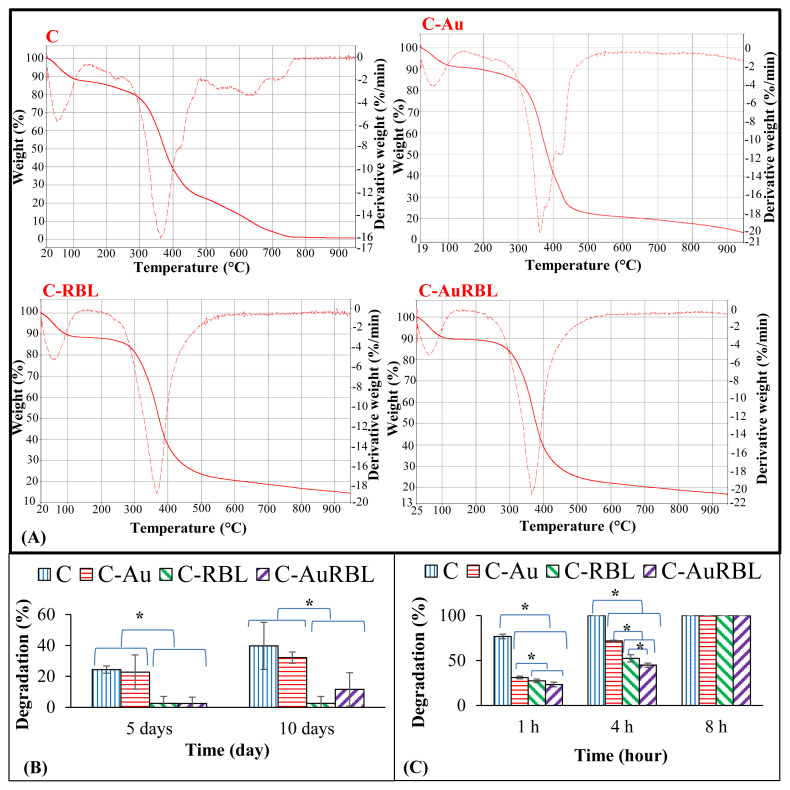
TGA thermograms and degradation results of different scaffold groups: (A) Thermograms, (B) hydrolytic, (C) enzymatic degradation test results. *: significant difference between groups for p < 0.05.

Figure 6 depicted the degradation profiles of scaffold groups either thermally or in an aqueous environment. In both analyses, the photochemically cross-linked scaffolds (C-RBL and C-AuRBL) retained their thermal and structural stability considerably. The weight losses, as well as their derivatives, are illustrated in the figures. Hence, 2 main degradation stages occurred in all groups. The first decomposition stage is due to the physisorbed or chemical water in the scaffolds and the second one is due to the melting or denaturation of collagen [36,37]. There were also third derivative peaks for C and C-Au groups at about 420 °C, which indicated that the degradation rate of these groups accelerated at this point. The fourth broad derivative peak emerging only for the C group signified that almost total combustion of collagen polymer was realized (decomposition or carbonization) to the end of 900 °C. Hence, it could be concluded here that both AuNPs and the cross-linking treatment seemed to enhance thermal stability. The trends resembled each other in hydrolytic as well as enzymatic degradation graphics (Figures 6B and 6C) and statistically significant results were obtained between cross-linked and uncross-linked groups for the incubation times. During 10 days of incubation in PBS buffer, C-RBL and C-AuRBL resisted the dissolution to a large extent (only 3%–12% degradation) as demonstrated in Figure 6B whereas all the groups were subjected to rapid enzymatic degradation (Figure 6C). The plain collagen (Group C) being the most vulnerable to ED, there occurred significant enhancement of resistance to ED after both S-AuNP incorporation in the scaffolds and RBL treatment as an analogy to thermal stability results.

**Figure 7:**
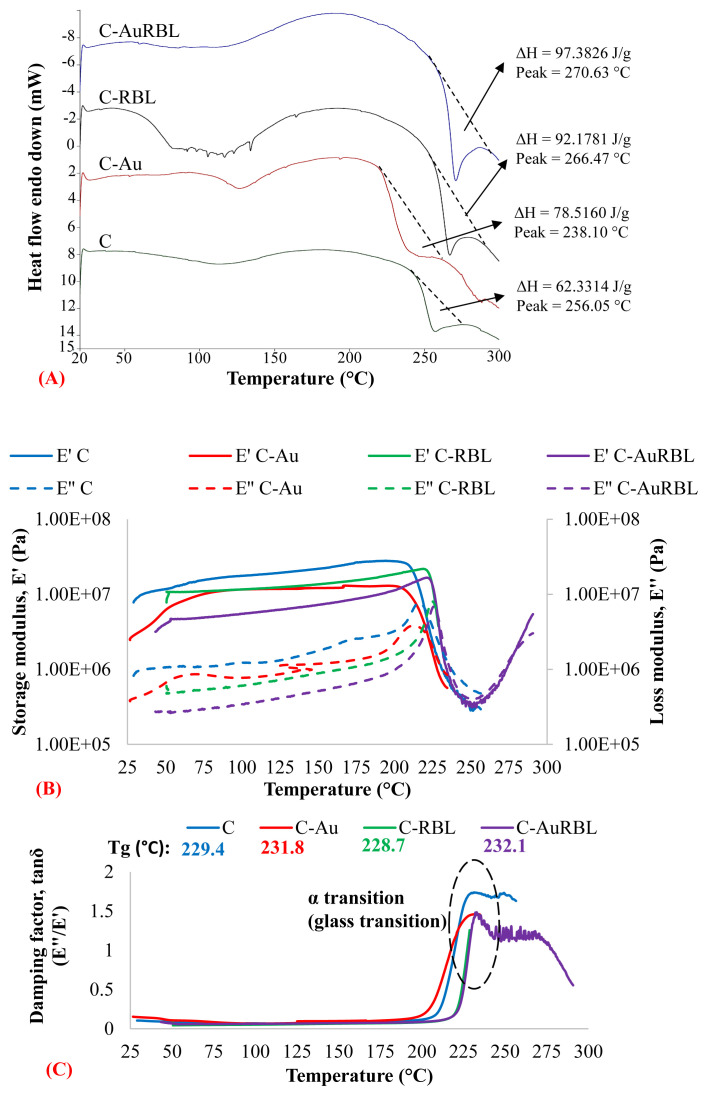
(A) DSC thermograms of scaffolds recorded during the first heating and the DMA thermomechanical plots demonstrating the changes of (B) storage modulus (E′), loss modulus (E″), and (C) the damping factor (tanδ) of scaffolds during temperature sweep.

The DSC curves of the first heating of test groups are presented in Figure 7A. The processes observed on the first heating run were considered irreversible because the curve recorded for the second heating run was smooth which is consistent with the findings in the literature [38]. Therefore, results for the second heating runs were not presented. The endothermic peaks originating from the transition of the triple helix structures of collagen molecules into random structures were not very distinct. The process is the breakage of intra- and intermolecular hydrogen bonds and the release of loosely bound water which usually occurs below 100 °C. In contrast, the endotherm with a peak temperature above 200 °C is due to the thermal degradation of polypeptide chains, and evaporation of residual and or strongly bound water [39]. The cross-linked groups (C-RBL and C-AuRBL) had significantly higher thermal degradation temperatures (T_td_). T_td_ values of the scaffold groups (C, C-Au, C-RBL, and C-AuRBL) were determined from the maximum transition points as 256.05 °C, 238.10 °C, 266.47 °C, and 270.63 °C, respectively. The increase in T_td_ values resulted from the higher cross-linking degrees of the collagen with photochemical treatment which in turn increases the thermal stability. It could also be seen that the enthalpy of the endotherm peaks increased for the cross-linked groups (ΔH = 92.1781 J/g for C-RBL and ΔH = 97.3826 J/g for C-AuRBL) compared to the plain collagen sponge (ΔH = 62.3314 J/g) and collagen sponge incorporated with S-AuNP (ΔH = 78.5160 J/g).

The effect of AuNPs and the RBL cross-linking process in the viscoelastic property of collagen scaffolds was studied over a temperature range of 25–300 °C and dramatic changes in storage (elastic) and loss modulus (viscous) were reported (Figure 7B). As compatible with the DSC results, until reaching the transition point of complete thermal degradation each group behaved predominantly in an elastic behavior (i.e. E′ > E″). Andonegi et al. [40] obtained very similar results with collagen films incorporated with zinc oxide nanoparticles along with their whole temperature range (−100 °C to 120 °C). The gradual increase in temperature resulted in the dehydration of scaffolds, thus making the collagen fibrils more compact and stiffer [41]. The α transition points, which are correlated with the glass transition temperature (T_g_), were detected from the tanδ peaks at 229–232 °C (Figure 7C). The temperatures for onset E′ and max E″ were also extrapolated for C, C-Au, C-RBL, and C-AuRBL groups as 210.9–216.4 °C, 209.2–212.4 °C, 221.1–224.2 °C, 225.3–225.4 °C, respectively. It could be inferred from these T_g_ values that while the addition of S-AuNP increased the α transition temperature slightly (≈ 3 °C) based on the max tanδ, the RBL cross-linking treatment increased the T_g_ values significantly (≈ 20 °C) obtained from E′-E″ plots. Well above 200 °C, the storage moduli of groups decreased sharply whereas loss moduli behaved conversely implying that the viscosity of scaffolds increased. The damping capability (i.e. tanδ) of uncross-linked and cross-linked groups followed a very similar trend until the threshold value of 200 °C, then the difference of tanδ between uncross-linked (≈ 0.5) and cross-linked (≈ 0.1) groups became significantly high at 210 °C. The same trend in the chitosan films impregnated with silver nanoparticles was reported in the previous study of Madian and Mohamed [42] and this was attributed to the increase of viscous property or the decrease of chain mobility in the polymer structure. It is obvious from the plots that the storage moduli of plain collagen scaffold are higher than other groups, which is indicative of its higher crystallinity [42].

### 3.3. Cell culture, cytotoxicity, and cell seeding studies

**Figure 8:**
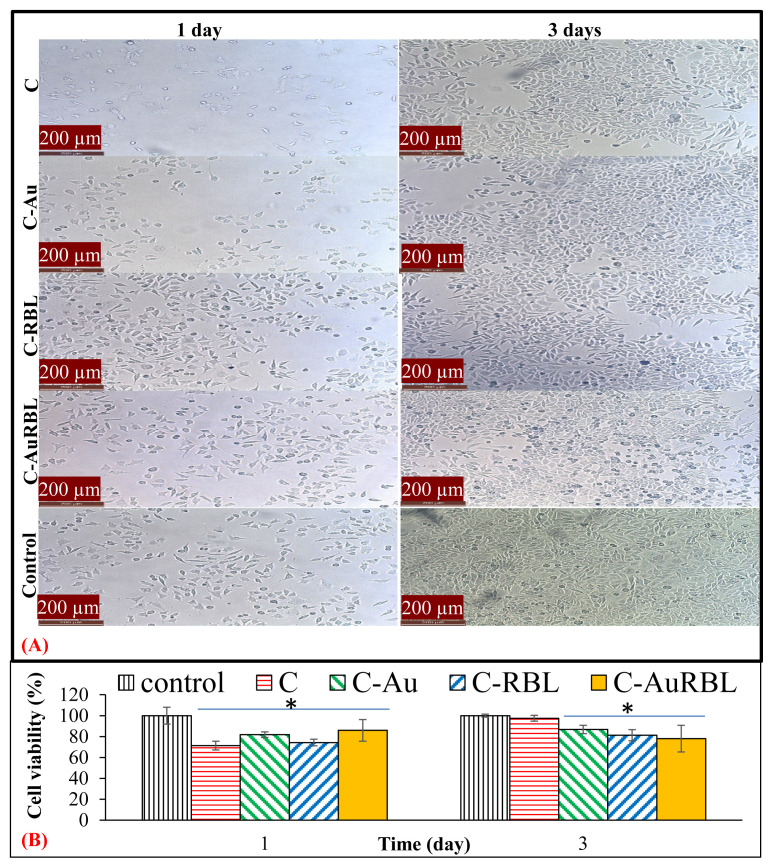
Cytotoxicity test results: (A) light microscopy imaging of L929 cell lines in 12 well-plates, (B) the cell viability results treated with the scaffold groups. *: significant difference from control for p < 0.05.

**Figure 9:**
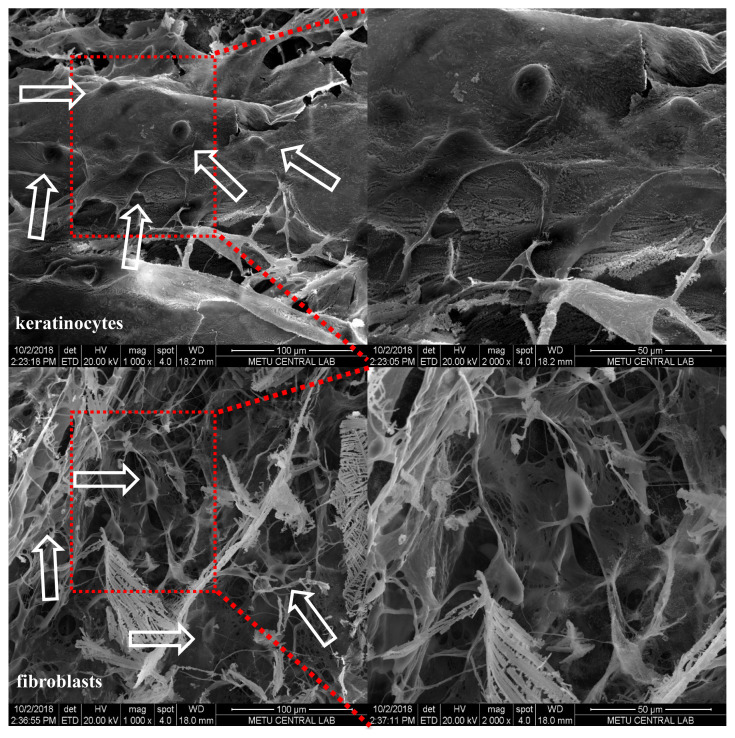
Morphology analysis of HEKa and L929 fibroblasts on C-AuRBL scaffolds: (A) SEM micrographs taken after 1-day incubation at different magnifications. Arrows indicate the cell locations.

The microscopic visualization of cells enabled a qualitative assessment of scaffold cytotoxicity (Figure 8A). The cytotoxicity of possible eluted scaffold fragments or any other released product (e.g., collagen, RB, and sericin capped-AuNPs, etc.) was examined by their effect on cell lysis and roundness. L929 cells gained their characteristic elongated morphology after 1 day of incubation period and they continued proliferating during 3 days of incubation (Figure 8A). As compared to the control group, the scaffold groups resulted in very small empty areas between cell colonies and the formation of round cells up to 5%–10%, which is an indication of slight cytotoxicity. RB remnants physically adsorbed on the scaffold were thought to be culpable for this slight cytotoxicity since the highest ratio of round cells (approx. 10%) was observed in the C-AuRBL group after 3 days of incubation. In parallel to visual observations of cells, quantitative MTT cell viability results supported the above findings as well (Figure 8B). While cell viabilities were in the range of 71%–86% after 1 day, they became 78%–98% after 7 days of incubation. When applying the test procedure, the specimens started floating in the media and did not come into direct contact with the cell monolayer; however, while discarding the specimens during experimentation steps (before microscopy observation and after the MTT assay), cell detachment occurred unintentionally, which might have caused underestimation of cell viability values. Cell adhesion, morphology changes, and migration on the C-AuRBL scaffold during 1 day of incubation were analyzed by SEM examinations (Figure 9). The healthy spindle-like and polygonal cell morphologies of L929 fibroblasts and HEKa, respectively, were confirmed with the micrographs (Figure 9). Both cells adhered to the scaffold surface conforming to the surface contours and started migrating to the deeper hollow regions.

The current study intended to explore the suitability and efficacy of RB molecules for collagen photothermal cross-linking treatment by laser irradiation in particular and to investigate the applicability of these scaffolds in skin tissue engineering applications. Overall, the cross-linked collagen scaffolds incorporated with sericin-capped AuNPs (C-AuRBL) had potency as skin tissue engineering constructs for their enhanced aqueous and thermal stability, viscoelastic properties, and quite porous morphology enabling a noncytotoxic environment for cells to adhere, proliferate and migrate. Future studies are directed toward confirming HEKA cell differentiation with characteristic cell surface differentiation markers and designing the C-AuRBL group as a reconstructed human epidermis (the so-called “in vitro RHE model”) with performance tests to evaluate its barrier function, morphogenesis, and so on.
